# Evaluation of the effect of guidelines to reduce intravenous potassium infusions in ICU patients

**DOI:** 10.1186/cc14442

**Published:** 2015-03-16

**Authors:** MC Law Min, RS Bourne, S Burd, M Stone

**Affiliations:** 1 Sheffield Teaching Hospitals, Sheffield, UK; 2 De Montfort University, Leicester, UK

## Introduction

The aim was to evaluate whether guidelines for intravenous (i.v.) potassium replacement improved plasma potassium homeostasis in ICU patients. Prompt and effective treatment of hypokalaemia is an important intervention in the ICU, but concentrated i.v. potassium solutions may cause serious harm if used inappropriately [1]. There were previously no formalised guidelines on i.v. potassium supplementation in the ICU at Sheffield Teaching Hospitals. Practice was reviewed and guidelines were introduced to improve patient safety, plasma potassium homeostasis and reduce i.v. potassium supplementation requirements.

## Methods

A before and after evaluation of plasma potassium homeostasis in ICU patients requiring i.v. potassium supplementation was conducted over a period of 8 months (August 2013 to May 2014). Patient data on plasma potassium levels, i.v. and oral potassium supplements administered were obtained from the clinical information system. Clinical appropriateness of i.v. potassium acetate prescriptions, fluid and chloride intake related to potassium infusions and cost linked to the guidelines were also compared pre/post implementation. Impact of the guidelines on nurses' practice was assessed using questionnaires.

## Results

Median i.v. potassium replacement dose per patient was significantly reduced in the post-guidelines group from 215 (IQR: 94; 485) to 80 (IQR: 40; 160) mmol; *P *< 0.001. Although the percentage time per group for patients who were hypokalaemic was less in the post group (18.2% vs. 14.8%), there was no difference in mean patient values (24.2 (20.3)% vs. 22.1 (17.5)%; *P *= 0.228). The duration of hyperkalaemia was increased. Prescribing of i.v. potassium acetate was not always appropriate. Median patient fluid-related dose was increased (107.5 (IQR: 47.1; 242.4) vs. 250 (IQR: 100; 600) ml; *P *< 0.001), whilst chloride doses were reduced (170.7 (IQR: 91.3; 438.3) vs. 110 (IQR: 55; 250) mmol; *P *< 0.009). Nurses were satisfied with the new practice, reporting it was safe, effective and clinically useful. However, compared with baseline practice, they perceived the guidelines as less effective and felt the workload was higher.

## Conclusion

Implementation of i.v. potassium replacement guidelines improved the use of i.v. potassium in the ICU by reducing the requirement for i.v. potassium supplementation and increasing the overall time patients spent without hypokalaemia. Whilst nursing staff found the guideline useful and felt it increased safe use of i.v. potassium, more work is needed to ensure nurse workload is not increased significantly.
